# First person – Capucine Januel

**DOI:** 10.1242/bio.062698

**Published:** 2026-06-22

## Abstract

First Person is a series of interviews with the first authors of a selection of papers published in Biology Open, helping researchers promote themselves alongside their papers. Capucine Januel is first author on ‘ Cat brains age like humans: Translating Time shows pet cats live to be natural models for human aging’, published in BiO. Capucine is a veterinary student in the lab of Christine Charvet at École Nationale Vétérinaire de Toulouse, France, investigating comparative neuroscience, brain aging and the use of veterinary medicine to better understand neurological diseases across species.

**Figure:**
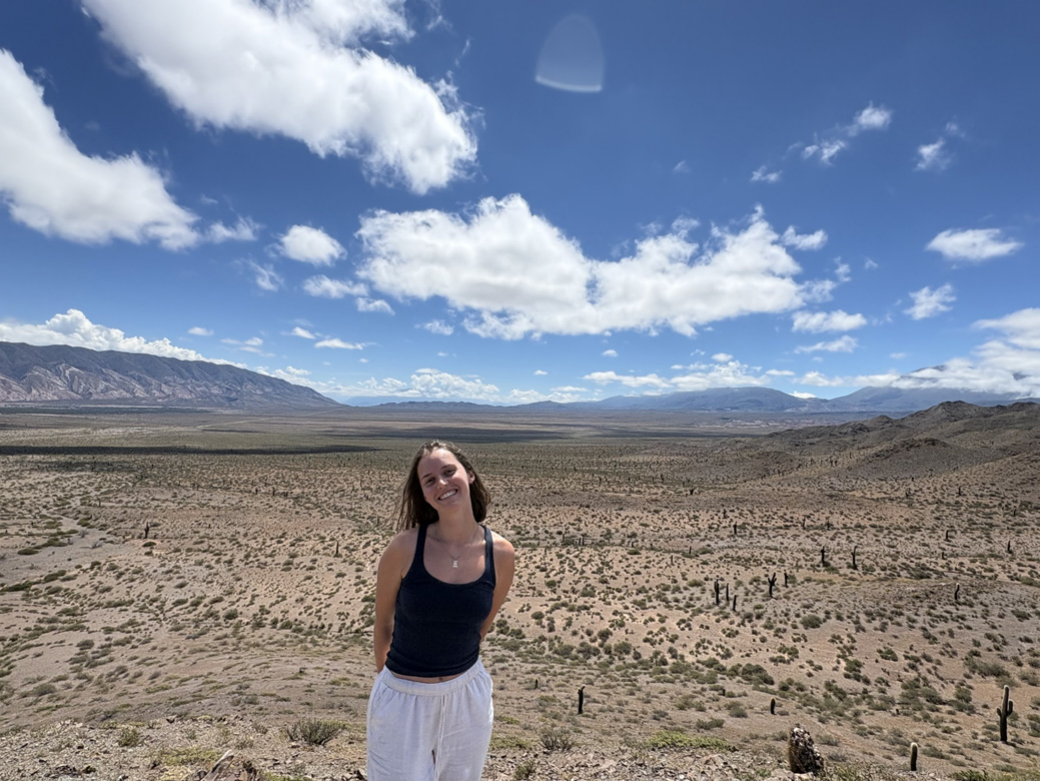
Capucine Januel

**Describe your scientific journey and your current research focus**

I am currently a veterinary student at École Nationale Vétérinaire de Toulouse in France, with a strong interest in neurology and comparative neuroscience. During my research experience at Auburn University, USA, I worked on the analysis of feline brain MRI scans to study age-related brain changes in cats and compare them with humans. This project allowed me to combine veterinary medicine, neuroimaging and aging research. I became especially interested in how naturally aging companion animals can help us better understand human neurological diseases. My long-term goal is to specialise in veterinary neurology while continuing to contribute to research.

**Who or what inspired you to become a scientist?**

I have always been fascinated by animals and medicine, but I became especially interested in research when I realised how much veterinary medicine can contribute to human health. The idea that studying naturally occurring diseases in animals could improve our understanding of human aging and neurology was very inspiring to me. Working with researchers from different scientific backgrounds also showed me how collaborative and creative science can be. I enjoy the process of asking questions, analysing data and trying to understand complex biological mechanisms.

We found that cats age in ways that are surprisingly similar to humans

**How would you explain the main finding of your paper?**

People often use simple formulas to compare cat and human ages, but aging is actually much more complex. In our study, we combined information from brain scans, blood tests and developmental milestones to compare aging across species. We found that cats age in ways that are surprisingly similar to humans, especially in the brain. Older cats naturally develop brain changes that resemble healthy human brain aging. Our results also suggest that pet cats can reach ages comparable to humans in their 80s, making them valuable natural models for studying aging.

…companion cats could become important natural models for studying brain aging and age-related neurological diseases

**What are the potential implications of this finding for your field of research?**

Our findings suggest that companion cats could become important natural models for studying brain aging and age-related neurological diseases. Unlike many laboratory animals, cats naturally share our environment and spontaneously develop age-related changes similar to humans. This could help researchers better understand healthy aging and diseases such as cognitive decline or neurodegeneration. More broadly, this work shows that veterinary and human medicine can benefit each other.

**Figure:**
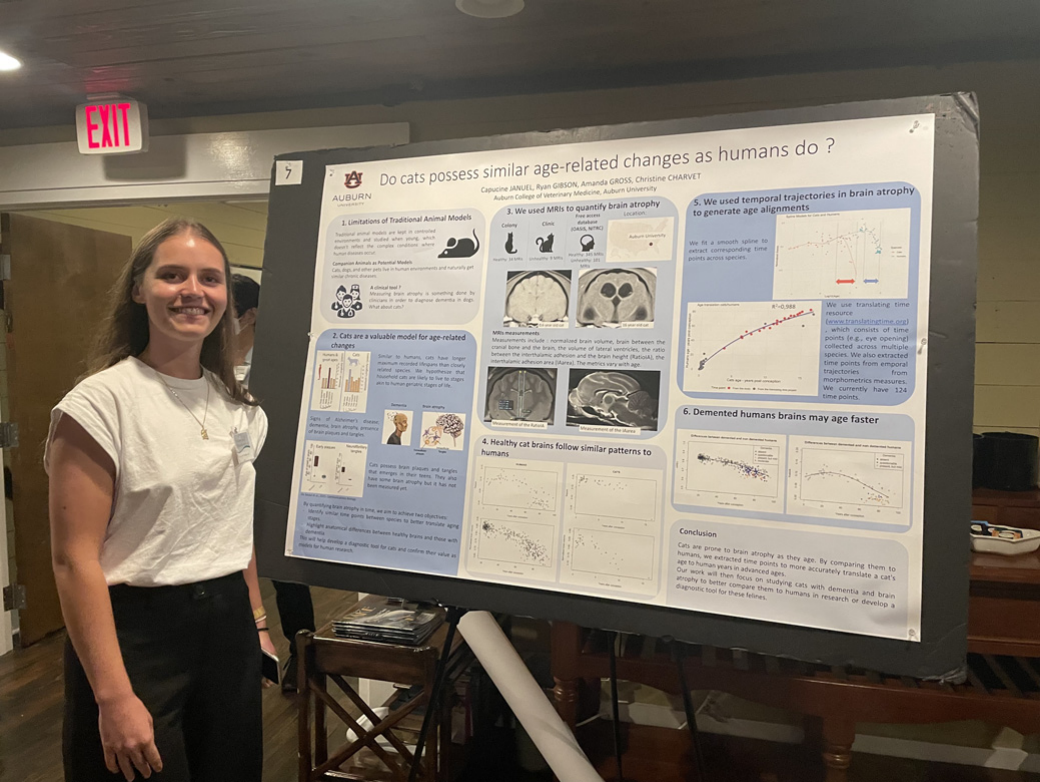
Presenting my research on feline brain MRI and aging for the first time at the annual Alabama neuroscientists’ retreat.

**Which part of this research project was the most rewarding?**

One of the most rewarding parts of the project was analysing MRI scans from cats of different ages and directly observing how the brain changes over time. It was exciting to see patterns that resembled human brain aging emerge from the data. I also really enjoyed working in an interdisciplinary team combining veterinary medicine, neuroscience and imaging analysis. As a veterinary student, contributing to a project with translational implications was especially motivating.

**What do you enjoy most about being an early-career researcher?**

I enjoy constantly learning new techniques and discovering different ways to approach scientific questions. Being an early-career researcher also means being exposed to many different fields and collaborations, which makes research very stimulating. I particularly appreciate the combination of clinical veterinary medicine and neuroscience research. It is exciting to feel that there are still many things to explore and understand.

**What piece of advice would you give to the next generation of researchers?**

Do not be afraid to explore interdisciplinary projects or step outside your comfort zone. Some of the most interesting discoveries happen at the intersection between different fields. I also think that you should never underestimate the value of collaboration and mentorship.

**What's next for you?**

I would like to pursue a career in veterinary neurology and if possible, continue working on neuroscience-related research projects. I am especially interested in neuroimaging, brain aging and translational medicine. In the future, I hope to combine clinical work with research to improve both animal and human health.
